# Do Genes Associated with Dyslexia of Chinese Characters Evolve Neutrally?

**DOI:** 10.3390/genes11060658

**Published:** 2020-06-17

**Authors:** Kumiko V. Nishiyama, Yoko Satta, Jun Gojobori

**Affiliations:** Department of Evolutionary Studies of Biosystems, SOKENDAI The Graduate University for Advanced Studies, Kanagawa 240-0193, Japan; nishiyama_kumiko@soken.ac.jp (K.V.N.); satta@soken.ac.jp (Y.S.)

**Keywords:** dyslexia, selective sweep, genetic hitchhiking, East Asian populations, population genetics, *nSL*, 2D SFS-based statistics

## Abstract

Dyslexia, or reading disability, is found to have a genetic basis, and several related genes have been reported. We investigated whether natural selection has acted on single nucleotide polymorphisms (SNPs) that were reported to be associated with risk/non-risk for the reading disability of Chinese characters. We applied recently developed 2D SFS-based statistics to SNP data of East Asian populations to examine whether there is any sign of selective sweep. While neutrality was not rejected for most SNPs, significant signs of selection were detected for two linkage disequilibrium (LD) regions containing the reported SNPs of *GNPTAB* and *DCDC2*. Furthermore, we searched for a selection target site among the SNPs in these LD regions, because a causal site is not necessarily a reported SNP but could instead be a tightly linked site. In both LD regions, we found candidate target sites, which may have an effect on expression regulation and have been selected, although which genes these SNPs affect remains unknown. Because most people were not engaged in reading until recently, it is unlikely that there has been selective pressure on reading ability itself. Consistent with this, our results suggest a possibility of genetic hitchhiking, whereby alleles of the reported SNPs may have increased in frequency together with the selected target, which could have functions for other genes and traits apart from reading ability.

## 1. Introduction

Dyslexia, or reading disability, is found to have a genetic basis [1,2,3,4,5,6], and has been observed among various writing systems [7]. It is usually diagnosed when an individual’s score falls below a cutoff in the normal distribution [6,8,9] using psychometric measures of reading and writing [1,3,10]. Continuously distributed traits, including reading ability, are considered to be polygenic traits [8,11]. Indeed, several genes have been reported to be related to dyslexia to date [1,2,3,4,5,6].

While genetic research on dyslexia was initially conducted in populations that use alphabetic languages, genetic factors of dyslexia in Chinese populations have been investigated in the last decade [12,13,14]. These studies found several single nucleotide polymorphisms (SNPs) whose risk/non-risk alleles were associated with some measures of reading (and writing) ability of Chinese characters (Table 1). Similar associations were found in preceding studies of populations using alphabetic languages (e.g., rs807724 on *DCDC2*), although alleles for risk or non-risk are not always the same between the populations studied, as found in rs4504469 on *KIAA0319* [15,16] and rs1091047 on *DCDC2* [12]. Among the reported SNPs, biological functions were experimentally investigated for rs3743205 on *DYX1C1* and rs1079727 on *DRD2* [17,18]. However, for most of the SNPs, their effects on biological function are unknown, and these SNPs themselves are not necessarily causal. Instead, the causal site may be a site that is tightly linked to a reported SNP [19].

From the perspective of human evolution, reading and writing are quite new activities, and have different histories to that of speaking. Writing systems were developed just a few thousand years ago and used by only a limited number of people before modern times; therefore, reading ability is unlikely to have been shaped by natural selection [28,29,30,31]. Dyslexia may be due to genetically based neurological variations that were not obstacles to humans until the introduction of public education in the 19th century [28]; before this time, dyslexic people would have lived without the reading difficulties/disadvantages that are present in modern society [9,32]. Based on this viewpoint, alleles related to reading ability are expected to be under neutral evolution. Otherwise, if natural selection has acted on such alleles, its target should be traits other than reading ability itself.

If natural selection has acted, at least two scenarios can be considered. The first scenario is proposed as the neuronal recycling hypothesis [29,33] or cultural neural reuse [31,34]. This is somewhat similar to the concept of exaptation, and explains the development of reading activity in humans as follows: An individual reuses a specific region of his/her brain, which functioned for something other than reading in the evolutionary past [29,31,33,34]. Natural selection can act on such prior functions, and in this case, a non-risk allele for dyslexia is expected to be the allele selected for the prior functions. The second scenario is pleiotropy, whereby a gene is involved in more than one function [35,36,37]. Thus, a locus could be selected not for functions related to reading itself but for other functions [38]; even alleles with risk for dyslexia could be selected if the risk alleles have an advantage for other functions.

Evolution of dyslexia-related genes has been investigated by comparing sequences of primates, which found a change in selective pressure on *ROBO1* after the divergence of the orangutan [39] and signs of positive selection on *KIAA0319* in the human lineage [38]. Some sites on *ROBO1*, *ROBO2*, and *CNTNAP2* showed signatures of selective sweeps within modern human populations, where the derived alleles significantly increased in frequency after the separation from archaic hominins, although they do not reach fixation [38]. As mentioned above, several sites on dyslexia-related genes were found to have risk/non-risk alleles associated with reading ability, although evolutionary analyses in these previous studies [38,39] did not focus on such alleles. The question in the present study is whether natural selection has acted on the alleles of SNPs that were reported to be risk/non-risk for reading ability. It is expected that there should not have been selective pressure on an individual’s reading ability. Moreover, it is more unlikely that alleles of the SNPs related to the reading ability of a certain writing system were selected especially for features of the writing system; the time for adaptation to a writing system to occur is probably insufficient [28,29,30,31].

Among various writing systems in the world, Chinese characters showed the earliest form around 1200 BCE, and have also been used at least once during history in other East Asian countries (e.g., Japan, Korea, and Vietnam), where spoken language systems are different from China [40]. Chinese characters have distinct features: Most characters are visually complex because they are compound characters, it contains semantic radicals, and thousands of characters exist [7,41]. Neurological studies showed that the brain areas involved in dyslexia are different between English and Chinese characters [42,43,44]. By examining East Asian populations, we investigated whether alleles of the SNPs found to be associated with reading ability had evolved neutrally or not. Although our focus was the reading ability of Chinese characters, we also considered that genes associated with dyslexia of Chinese characters could be selected for their other functions as in pleiotropy.

We performed neutrality tests on the SNPs associated with the reading/writing ability of Chinese characters (Table 1). Because each type of neutrality test would have its suitable time scale to detect the signature of selection [45], we used two different types of summary statistics: Number of segregating sites by length (*nSL*) [46] and 2D SFS [47,48], which are based on extended haplotype homozygosity (EHH) and the site frequency spectrum (SFS), respectively. EHH-based statistics, such as *nSL*, are powerful at detecting signs of recent selective sweep, where linkage disequilibrium (LD) is expected to be relatively maintained [45]. Meanwhile, 2D SFS-based statistics can detect sweep signals in regions that have experienced recombination events over time and result in being with short LD. We focused on derived alleles of the SNPs regardless of whether they are risk or non-risk for reading ability, while considering the case of selection for pleiotropy. We also considered SNPs that were tightly linked to the SNPs associated with reading/writing ability (Table 1), because they also could be causal for reading ability or have other functional effects, and therefore could be selection targets. In such cases, a reported SNP may be considered a hitchhiker of a tightly linked SNP that is under selection. To search for the selection targets, we analyzed in detail the LD regions that contain the candidate SNPs under selection.

## 2. Materials and Methods

### 2.1. Examined SNPs

We focused on 15 SNPs that were found to be associated with dyslexia of Chinese characters in previous studies (Table 1). Hereafter, these SNPs will be referred to as “core SNPs”.

### 2.2. Study Populations

We examined East Asian populations, expecting if natural selection has acted on genes associated with dyslexia of Chinese characters, the signature would be seen in these populations. At present, publicly available data of these populations were East Asian populations (EAS) in the 1000 Genomes Project phase 3 (1 KG) [49] and the Korean population from The Personal Genome Project Korea (KPGP) [50,51]. We used them as study populations.

We downloaded 1 KG and KPGP data from ftp://ftp.1000genomes.ebi.ac.uk/vol1/ftp/release/20130502/, and from ftp://biodisk.org/Release/KPGP/KPGP_Data_2017_Release_Candidate/WGS_VCF_89_KOREAN_JOINT_CALL/, respectively. 1 KG was comprised of 2504 individuals from 26 global populations, and KPGP was comprised of 88 individuals (one sample of KPGP-00349 was removed because it was reported as a non-Korean sample on the ftp site). For KPGP, only SNP data with a filter status of “PASS” were used.

The unphased KPGP data was phased using Eagle2 [52]. As the reference panel for phasing, we used 1 KG after excluding singleton and duplicated SNPs. The imputation of missing genotypes was not employed.

#### 2.2.1. Study Populations for *nSL*

After the phasing procedure, we merged KPGP with 1 KG. The merged data includes only sites that exist in both 1 KG and KPGP. From the merged data, we extracted data of individuals in EAS and KPGP. The extracted data (EAS-KPGP, hereafter) was comprised of 594 individuals.

#### 2.2.2. Study Populations for 2D SFS-Based Statistics

2D SFS-based statistics [47,48] require plenty of phased SNPs and are sensitive to singletons. The phasing and merging procedures for EAS-KPGP described above led to a reduced number of SNPs in the data and are expected to be deficient in rare SNPs, because the procedures restricted the merged data to contain only sites existing in both 1 KG and KPGP. For this reason, EAS-KPGP would be inadequate for 2D SFS-based statistics. Therefore, we used only EAS (504 individuals) for the 2D SFS-based statistics. We used biallelic SNP data, with information of ancestral states and without missing genotypes.

### 2.3. nSL

We used *nSL* [46] as a summary statistic for a neutrality test based on EHH. We applied *nSL* to EAS-KPGP, using the *selscan* program [53]. For calculation of the *nSL* values, only biallelic SNPs with a minor allele frequency ≥ 0.01 were retained. SNPs with missing genotypes and in the major histocompatibility complex (MHC) region (chr6: 28,477,797–33,448,354 of GRCh37) were not used. We referred to information in 1 KG for ancestral states of each SNP. The EHH decay cutoff was extended by setting the program option of --max-extend-nsl as 1500, which allowed more accurate *nSL* computation than the default of 100. The total number of SNPs in the data was 6,143,039. *nSL* values were normalized in 100 frequency bins, which is the default setting. One-tailed *p*-values were obtained to check neutrality on derived alleles.

### 2.4. 2D SFS-Based Statistics

#### 2.4.1. Overview of 2D SFS-Based Statistics

In order to examine the neutrality of core SNPs and the surrounding regions, we conducted the 2D SFS-based statistics recently developed by Fujito et al. [47] and Satta et al. [48]. These statistics measure the intra-allelic variability (IAV) [54,55], or the level of polymorphism within haplotypes carrying the derived allele of a focal site (core site). Among the several statistics related to 2D SFS, we used two for the present study: $F_{c}$ and $G_{c0}$. The full derivation and equations are presented in Fujito et al. [47] and Satta et al. [48], and a general overview will be presented here.

We considered segregating sites in a region with high LD, which contains a core site. We assumed n chromosomes sampled from a single diploid population. The n samples are divided into two groups: The derived allele group (D group) that carries the derived allele of the core site, and the ancestral allele group (A group) that carries the ancestral allele. The size of the D group is m (1≤m<n) and that of the A group is n−m. At a certain site other than the core site in the region, the number of derived alleles in the D group is described as i (0≤i≤m), and the number of derived alleles in the A group as j (0≤j≤n−m). Then, the 2D SFS of each site is represented as the matrix $\left\{\varphi_{i,\;j}\right\}$.

The SFS for the entire sample is expressed as:

(1) $$\xi_{k}=\sum_{i=0}^{k}\varphi_{i,k-i}\;\mathrm{for}\;1\leq k<n,\;\mathrm{where}\;k=i+j,$$

corresponding to Equation (1a) in Satta et al. [48], and analogously, the SFS for the D group is expressed as:

(2) $$\zeta_{i}=\sum_{j=0}^{n-m}\varphi_{i,j}\;\mathrm{for}\;1\leq i<m,$$

corresponding to Equation (1b) in Satta et al. [48].

The statistics of $F_{c}$ measure the ratio of the amount of mutations in the D group to that in the entire sample, using only mutations younger than the derived allele at the core site [47]. The number of derived alleles at a site implies the age of the mutation: A large number (high derived allele frequency) is expected to be an old mutation whereas a small number (low derived allele frequency) suggests a young mutation [47,55,56,57]. To exclude mutations older than the mutation on the core site, which should be shared by both the D and A group, the $F_{c}$ statistic uses “frequency class(es)” based on the scaled mutation rate $\theta =4N_{e}u$, where $N_{e}$ is the effective population size and u is the mutation rate per region per generation. From $E\left\{\xi_{k}\right\}=\theta /k$ [58], each frequency class is described as class 1 with $E\left\{\xi_{1}\right\}=\theta$, class 2 with $E\left\{\xi_{2}+\xi_{3}\right\}=5\theta /6$, class 3 with $E\left\{\sum_{k=4}^{9}\xi_{k}\right\}\approx \theta$, class 4 with $E\left\{\sum_{k=10}^{25}\xi_{k}\right\}\approx \theta$, class 5 with $E\left\{\sum_{k=26}^{68}\xi_{k}\right\}\approx \theta$, and so on. The $F_{c}$ statistic is expressed as:

(3) $$F_{c}=\frac{\Sigma i\varphi_{i,j}}{\Sigma \left(i+j\right)\varphi_{i,j}}\;\mathrm{for}\;i+j\leq k_{m}<m,$$

corresponding to Equation (4) in Fujito et al. [47]; Equation (2) in Satta et al. [48], where $k_{m}$ is the upper bound number of derived alleles of a frequency class that is one class lower (i.e., younger) than the class containing m.

The statistics of $G_{c0}$ compute the average number of derived alleles per segregating site only observed in the D group, excluding polymorphisms caused by recombination between the D and A group [48]. The $G_{c0}$ statistic is expressed as:

(4) $$G_{c0}=\frac{\sum_{i=1}^{m-1}i\varphi_{i,0}}{\sum_{i=1}^{m-1}\varphi_{i,0}},$$

corresponding to Equation (7) in Satta et al. [48].

In both statistics, the values are expected to be relatively small under selective sweep.

#### 2.4.2. Simulations

To obtain *p*-values of $F_{c}$ and $G_{c0}$, we performed simulations by *ms* [59]. We assumed neutrality without recombination and with the demographic model of Schaffner et al. [60], following Fujito et al. [47] and Satta et al. [48]. We sampled 30,000 replications, each of which contained a core site with a similar derived allele frequency to a focal SNP (e.g., core SNP). The derived allele frequency for core sites in simulations ranged within one standard deviation of a binomial distribution, as $fr\pm \sqrt{\frac{fr\left(1-fr\right)}{n}}$, where fr is m/n of a focal SNP. From the 30,000 replications, we described null distributions of $F_{c}$ and $G_{c0}$, and obtained the *p*-values of $F_{c}$ and $G_{c0}$ of a focal SNP. We confirmed that 30,000 replications is large enough to obtain stable results.

#### 2.4.3. Screening of the Candidate Core Regions under Selective Sweep

Screening for further analysis was carried out to examine whether there is a sign of selective sweep in each high LD region containing a core SNP (“core region”). We collected neighboring SNPs that had $r^{2}$ with the core SNP ≥ 0.75 (“linked SNPs”, hereafter) within a 0.5 Mb region in both directions of the core SNP. We then defined the boundaries of each core region by the linked SNPs that were located in the most upstream and downstream positions (Figure S1). Note that $r^{2}$ also becomes large when a derived allele at the core SNP is linked to ancestral alleles in the linked SNPs and vice versa (ancestral allele at core SNP linked to derived alleles in linked SNPs). We did not use SNPs that displayed this pattern for determining boundaries of the core regions. For each core SNP in its core region, we applied the $F_{c}$ statistic, which detects the sweep signal by quantifying the amount of mutations in the D group after the emergence of a core SNP.

#### 2.4.4. Searching for the Target Site of Natural Selection (“Target Site”)

After identifying candidate core regions under the selective sweep from screening (where the $F_{c}$ value of the core SNP has *p* < 0.1), we further analyzed these regions in detail. Here, the aim was to search for the target site of natural selection (“target site”) by comparing the level of polymorphism around each of the candidate SNPs (core SNP and its linked SNPs) in the core region. It is expected that the level of polymorphism in the D group would be low around the target site due to selective sweep, and this level would increase with distance from the target site. Under this expectation, we used the $G_{c0}\;$ statistic to examine the average amount of mutations within the D group of each candidate SNP in order to identify the target site.

In order to use the $G_{c0}\;$ statistic, a surrounding region of each candidate SNP was defined. Firstly, within the core region, we calculated $G_{c0}$ for all possible region lengths containing the specific candidate SNP. Next, we selected the region with the smallest $G_{c0}$ value (“smallest region”). For statistical reliability, each region was set to contain at least 100 SNPs. If more than one region had the same smallest $G_{c0}$ value, we selected the region containing the largest number of SNPs.

We applied this procedure to all candidate SNPs within the core region. The length of the smallest region varied among candidate SNPs, and because $G_{c0}$ values were affected by the region length or the number of SNPs in the region, we could not directly compare the $G_{c0}$ values of the smallest region of all candidate SNPs. Thus, we examined how unlikely the $G_{c0}$ value of each candidate SNP was to be produced under neutrality, by converting the $G_{c0}$ values into the *p*-values obtained from simulations. We compared these *p*-values with each other.

## 3. Results

### 3.1. nSL

We removed SNPs containing missing genotypes because *selscan* required data without missing genotypes for *nSL*. We could not obtain *nSL* for the core SNP of rs28366021 on *KIAA0319L* because it contained 14 missing genotypes in KPGP. Instead of rs28366021, we examined a neighboring SNP (rs11264175) located 7.5 kb downstream from the core SNP. We used this neighboring SNP because the $r^{2}$ value of rs11264175 with rs28366021 was the highest ($r^{2}$ = 0.957) in the data when the 14 samples with missing genotypes were excluded.

Moreover, *nSL* could not be properly calculated for the core SNP of rs2255526 on *DIP2A*. This SNP was located at the edge of chromosome 21, and extended haplotypes reached the end of the chromosome before EHH decayed entirely.

We checked normalized *nSL* and their *p*-values of the core SNPs (rs11264175 representative of rs28366021 on *KIAA0319L*), except rs2255526 on *DIP2A*. For all 14 SNPs, normalized *nSL* values were not significant (*p* ≥ 0.01 for all; Table 2). Therefore, *nSL* did not detect any signatures of positive selection for any of the core SNPs.

### 3.2. 2D SFS-Based Statistics

For the 2D SFS-based statistics, we used two steps. First, we conducted screening using the $F_{c}$ statistic to check whether a high LD region containing a core SNP (core region) could be under selective sweep. Second, we used the $G_{c0}$ statistic to analyze the core regions that passed the screening, in order to search for the target site of natural selection.

#### 3.2.1. Screening of the Candidate Core Regions under Selective Sweep

To apply the $F_{c}$ statistic to each core SNP, we needed to determine its core region. To do so, we extracted its “linked SNPs” ($r^{2}>$ 0.75) (see the methods section; Figure S1). However, we could not define the core region for rs2074130 on *DOCK4* because no linked SNPs were identified. This meant that $F_{c}$ statistic could not be applied to this SNP. Thus, the SNP was omitted from subsequent analyses including the $F_{c}$ statistic. Based on the absence of an LD region, we inferred that the derived allele of rs2074130 was not under positive selection, because if selection had acted, then the derived allele should at least have some extent of LD as a signature of the genetic hitchhiking.

At this stage of the screening, we could not determine whether the target site of selection was the core SNP or one of its linked SNPs. Thus, we considered both a core SNP and the linked SNPs in a core region as candidates for the target site. The number of derived alleles of linked SNPs should be similar to that of the core SNP, and therefore, the age of linked SNPs is expected to be similar to that of the core SNP. However, even if linked SNPs showed a similar number of derived alleles in a local population, such as EAS, they could show a different number from the core SNP when looking at the global population. The level of polymorphism should be different between the core SNP and such linked SNPs, due to the difference in age. For such linked SNPs, it is inappropriate to apply the statistic to its core SNP.

For this reason, we checked the global derived allele count (number of derived alleles in the entire population in 1 KG) of a core SNP and its linked SNPs, in addition to the count in EAS. Next, each SNP was classified into a “frequency class” (see the methods section). We found that three core SNPs (rs4535189 on *ROBO1*, rs1091047 on *DCDC2*, and rs3743205 on *DYX1C1*) had some linked SNPs with global derived allele counts smaller than that of their core SNPs, and that these linked SNPs were classified into lower (i.e., younger) frequency classes than their core SNPs. The global derived allele count of rs4535189 on *ROBO1* is 2280 and belonged to frequency class 9; in the core region, 16 of the 23 linked SNPs were classified into the same class 9 as the core SNP, but 7 linked SNPs were classified into class 8. Similarly, the global derived allele count of rs1091047 on *DCDC2* was 3871 and belonged to class 10; 7 of the 16 linked SNPs were also classified into class 10, but 9 were classified into class 9. Moreover, the global derived allele count of rs3743205 on *DYX1C1* was 517 and classified into class 8, whereas the classes of the 97 linked SNPs varied: 7 were classified into a class 7, 87 into class 6, and 3 into class 5. No linked SNPs were classified into the same class 8 as the core SNP.

In each of these three cases, in addition to the core SNPs, we analyzed one linked SNP in a younger class, because these linked SNPs should have different evolutionary depths and therefore different polymorphism levels from their core SNPs. For each of the three cases, among the several linked SNPs, we selected a linked SNP that showed the smallest derived allele count as the “younger SNP”: rs73129039 (global derived allele count = 1214 and frequency class 8) on *ROBO1*, rs3789228 (global derived allele count = 2583 and frequency class 9) on *DCDC2*, and rs79024225 (global derived allele count = 31 and frequency class 5) on *DYX1C1*.

We also found that some linked SNPs were classified into a globally older frequency class than their core SNP. However, we ignored such cases. The extent of polymorphism at an “older SNP” should be greater than that at a core SNP due to the difference in age. Although the $F_{c}$ value is expected to be small under selective sweep, the $F_{c}$ value at the “older SNP” cannot be smaller than that at the core SNP. Therefore, we did not examine older SNPs in subsequent analyses.

We screened core regions for detailed analysis. The $F_{c}$ statistic was applied to the 14 core SNPs and the 3 younger SNPs to identify the regions suspected to have experienced selective sweep, using statistical significance of α = 0.1. The *p*-values were obtained from simulations (Table 3), and two SNPs remained after this screening: rs17031962 on *GNPTAB* (*p* = 0.038) and rs3789228 (younger SNP for rs1091047) on *DCDC2* (*p* = 0.068).

#### 3.2.2. Searching for the Target Site of Natural Selection

On the two core regions that contained SNPs that passed screening (rs17031962 on *GNPTAB* and rs3789228 on *DCDC2*), we searched for the target site of natural selection using $G_{c0}$ (see the methods section).

##### rs17031962 on *GNPTAB*

The derived allele count at rs17031962 is 296 out of 1008 chromosomes in EAS. The reported risk allele is the ancestral allele [23]. The core region of rs17031962 is approximately 137 kb long (chr12: 102,096,776–102,233,579 of GRCh37) and contains two genes other than *GNPTAB*: *CHPT1* (partial) and *SYCP3*. *CHPT1* encodes cholinephosphotransferase [61], and *SYCP3* encodes a component of the synaptonemal complex, which is involved in the pairing and crossover of homologous chromosomes during meiosis [62]. We found that the core region contained 50 linked SNPs in the same global frequency class as rs17031962.

We found one possible phasing error for one of the SNPs (rs78494298). The derived allele count at rs78494298 was 15, and only 14 alleles were linked to the derived allele at rs17031962 (core SNP). For the calculation of 2D SFS, this was counted as $\varphi_{14,1}$. For sample HG00707, one of the two chromosomes carried the derived allele at rs17031962 and the ancestral allele at rs78494298. Conversely, the other chromosome carried the ancestral allele at rs17031962 and the derived allele at rs78494298; this is the cause of $\varphi_{14,1}$ at rs78494298. This pattern is not likely caused by recombination because surrounding SNPs did not display evidence of any cross-over event (Figure S2), and supports the possibility of a phasing error. Because the $G_{c0}$ statistic counts only $\varphi_{i,0}$ (and therefore ignoring $\varphi_{14,1}$), the state at rs78494298 results in a smaller $G_{c0}$ value and *p*-value than the case of $\varphi_{15,0}$, where the possible phasing error was corrected. We therefore altered the present (default) state of $\varphi_{i,j}\;$ at rs78494298 to $\varphi_{15,0}$. Regardless of whether this is a true phasing error or not, this manipulation provides an even more conservation approach, compared to the default state, for calculating $G_{c0}$.

Among the linked SNPs, we found that three consecutive SNPs (rs557004549, rs183736467, rs188452374) showed different patterns from the other linked SNPs (Figure S3). These three SNPs are completely linked to each other; some of their D group seemed to be linked to the A group of the core SNP and other linked SNPs, and vice versa. If haplotypes with the derived allele at these three SNPs were selected for, then LD is not expected to break down immediately. Therefore, we removed these three SNPs from subsequent analysis, assuming that none of them would be the target site. Subsequently, the number of the candidate SNPs became 48 (the core SNP and 47 linked SNPs).

**Figure 1:**
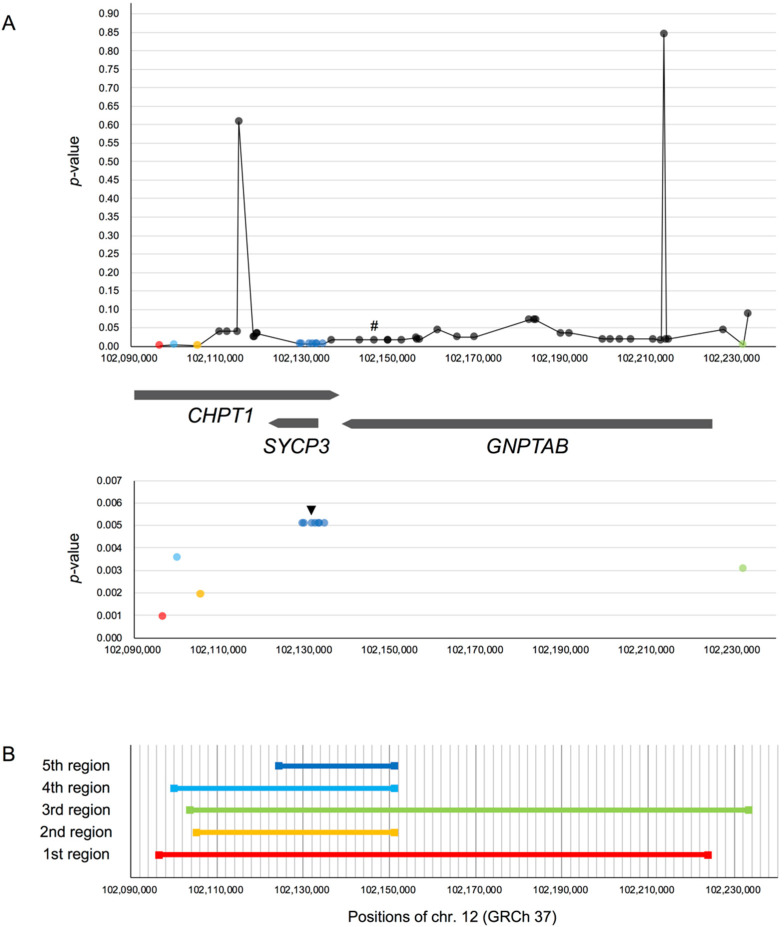
(**A**) Top: *p*-values of $G_{c0}$ for 48 candidate SNPs in the core region of rs17031962 on *GNPTAB*. Each dot represents a candidate SNP. The core SNP is indicated by “#”. Colored dots other than black indicate the 12 SNPs with *p* < 0.01. SNPs with the same *p*-value and smallest region are indicated in the same color. Positions of the three genes in the core region are illustrated as thick lines underneath. Bottom: The same plot showing only the SNPs with *p* < 0.01. The possible target site is indicated by a black arrow. (**B**) The lengths and positions of the smallest regions of the SNPs with *p* < 0.01. The regions are numbered according to the ascending order of the *p*-value. The color of the regions corresponds to the dot color in (**A**).

For each of the 48 candidate SNPs in the core region of rs17031962, we selected the region with the smallest $G_{c0}$ value, and obtained *p*-values from simulations (Figure 1A). Among them, 12 SNPs were statistically significant (*p* < 0.01; Figure 1A bottom). SNPs that overlapped in the same “smallest region” and shared the same *p*-values were grouped together into the same region. We identified five regions that contained significant SNPs; these regions were numbered according to the ascending order of *p*-values (Figure 1B).

The range of the smallest region for each candidate SNP, as well as the *p*-value, may provide insights into the target site. The candidate SNP of the first region (*p* = 0.0009) was one of the core region boundaries, and that of the third region (*p* = 0.0031) was located close to the other core region boundary, where LD seemingly began to break down ($r^{2}$ values for the first and third region are 0.934 and 0.892, respectively; Figure S1). The first region covers almost the entire core region, where the average amount of mutations in the D group (i.e., $G_{c0}$ value) was the smallest. Shorter regions with this candidate SNP had higher $G_{c0}$ values, indicating that the average amount of mutations in the area around this candidate SNP is high; this contradicts the expectation that the level of polymorphism around the target site is small. Thus, we do not consider the candidate SNP of the first region to be the target site. This also applied for the candidate SNP of the third region. Furthermore, while the second and fourth regions overlapped with the fifth region (Figure 1B), when we investigated shorter regions that covered the candidate SNP in the second (or fourth) region but not that of the fifth region, we found higher $G_{c0}$ values. From these observations, we considered that the fifth region may hold the target site, although the *p*-value of the SNPs in the fifth region (*p* = 0.0051) is the highest among the significant SNPs.

The candidate SNPs in the fifth region were located in *SYCP3* and its upstream region. To elucidate the possible biological trait under selection, we investigated the functional significance of the SNPs by checking the Ensembl Variant Effect Predictor (VEP) [63] for GRCh37. We found a candidate SNP in the fifth region (rs3751248) located on an open chromatin region; this SNP may have biological functions, possibly expression regulation, and the genotype difference may have different traits that affect individual fitness. Thus, we inferred that this SNP could be the target site.

##### rs3789228 on DCDC2, as the Younger SNP of rs1091047

rs3789228 is the “younger SNP” of rs1091047 on *DCDC2*. The number of derived alleles of rs3789228 (younger SNP) is 782 out of 1008 chromosomes in EAS, while that of rs1091047 (core SNP) is 824. The reported risk allele of the core SNP is the ancestral allele [12]. For this detailed analysis, we re-extracted linked SNPs of rs3789228. Almost all linked SNPs were clustered together. However, one linked SNP was located 38 kb from the cluster and thus removed from analysis as it is not likely to be the target site. Then, 20 linked SNPs in the same frequency class as rs3789228 (class 9) were collected. The core region of the younger SNP was ~43 kb long (chr6: 24,255,044–24,297,900 of GRCh37).

**Figure 2:**
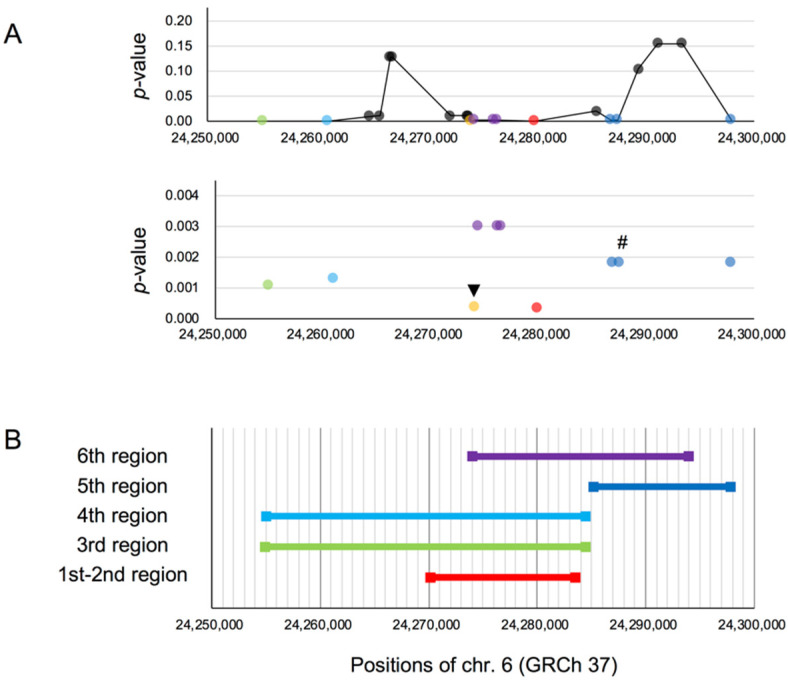
(**A**) Top: *p*-values of $G_{c0}$ for 21 candidate SNPs in the core region of rs3789228 on *DCDC2*. Each dot represents a candidate SNP. Colored dots other than black indicate SNPs with *p* < 0.01. SNPs with the same *p*-value and smallest region are indicated in the same color. Bottom: The same plot showing only the SNPs with *p* < 0.01. The target site is indicated by a black arrow. The younger SNP is indicated by “#”. (**B**) The lengths and the positions of the smallest regions of the SNPs with *p* < 0.01. The regions are numbered according to the ascending order of *p*-value. “1st-2nd region” indicates the smallest region containing both the top and the second SNP, shown as red and orange dots in (**A**), respectively, and overlapped in the same smallest region. The colors of the other regions correspond to the dot colors in (**A**).

For each of the 21 candidate SNPs (the younger SNP and 20 linked SNPs) in the core region, we selected the region with the smallest $G_{c0}$ and obtained the *p*-value for these $G_{c0}$ values by simulations (Figure 2A). Among them, 10 SNPs were significant (*p* < 0.01). The top SNP (*p* = 0.0003) and the second SNP (*p* = 0.0004) shared the same smallest region and were grouped together as the first to second region. We also grouped other SNPs together that were in the same smallest region and with the same *p*-value. In total, five regions were detected (Figure 2B), which we numbered according to the ascending order of the *p*-value.

Only the top and second SNPs showed *p* < 0.001. In a similar fashion to our other case (rs17031962 on *GNPTAB*), the first to second region was overlapped with both the third and fourth regions, and partially overlapped with the sixth region. Based on this, we considered that either the top or second SNP may be the target site. On VEP [63] for GRCh37, we found that the second SNP (rs12055879) and a single SNP in the sixth region (rs807700) were in both the enhancer region and CTCF binding sites, which may affect expression regulation. Considering the *p*-value, we inferred that the target site could be the second SNP.

## 4. Discussion

In order to investigate whether natural selection has acted on the core SNPs of interest, we conducted two types of neutrality tests on the derived alleles: *nSL* (as an EHH-based test) and 2D SFS-based statistics. For most of the core SNPs, neither statistics detected any signatures of selective sweep, thus neutrality was not rejected. Previous studies found signs of natural selection on dyslexia-related genes by phylogenetic analyses [38,39]. A significant increase of derived allele frequencies were reported in some sites on dyslexia-related genes in modern human populations [38]. While attempts to detect signatures of natural selection on dyslexia-related genes among modern human populations have been performed, our study focused on the SNPs that were reported to be associated with risk/non-risk for some traits related to an individual’s reading ability in one of the writing systems. Because most people were not engaged in reading and writing until recently [28,29,30,31], the genetic variations that our study focused on were unlikely to be maintained by natural selection, which is consistent with our results. Signs of acting natural selection were found on some alleles associated with autism spectrum disorder and schizophrenia [64,65]. Different from such traits, dyslexic traits should have been veiled until modern times. So, selective pressure on cognitive functions could be different between reading/writing and other traits. Nevertheless, the 2D SFS-based statistics suggested that two core regions could be under selective sweep. Because the selection target could be an SNP linked to a core SNP, we searched for the target site in these two exceptional cases.

The first case is the core region of rs17031962 on *GNPTAB*. The derived allele of this core SNP is the non-risk type [23]. In addition to *GNPTAB*, this region also contains genes of *CHPT1* (partial) and *SYCP3*. We searched for the target site using the $G_{c0}$ statistic and concluded that the target site could be an SNP (rs3751248) in one of the smallest regions with *p <* 0.01 (the fifth region), because it is located in an open chromatin region. However, even if this SNP has some biological function, it is still unknown which trait is affected. There are two possible scenarios where natural selection has acted on this SNP. The first scenario is the selection for the prior functions explained by the neuronal recycling hypothesis and cultural neural reuse [29,31,33,34]. In this scenario, the derived allele may have been selected for a prior function, and therefore, the derived allele was identified as the non-risk allele for the reading ability of Chinese characters. The second scenario is pleiotropy, which should also be considered. Although the core SNP was associated with dyslexia of Chinese characters [23], *GNPTAB* has been found to be related to stuttering [23,66,67]. Beyond functions related to language, this gene is involved in tagging for transport of lysosomal enzymes [66,67,68]. In addition, Ebola virus was recently reported to utilize *GNPTAB* for efficient infection [69]. If rs3751248, which we speculate to be the target site in this region, did not affect reading ability but instead some other function involving *GNPTAB*, then pleiotropy would explain this situation. However, it is unknown which gene is affected by a mutation on the target site (rs3751248). Because this SNP (rs3751248) is located in an open chromatin region, neither of the two scenarios can explain the case whereby the target site has a functional effect on genes other than *GNPTAB*. In such a case, our findings may be attributed to genetic hitchhiking, where alleles in dyslexia-related genes may increase their frequency together with the linked target site, which could have functions for other genes and traits other than reading ability. Thus, we consider this third scenario based on our results, and there may be other scenarios; however, it remains unclear which scenario actually occurred because of the current lack of understanding about the effect of mutations on the target site.

Although we focused on and analyzed only East Asian populations in this study, it may be valuable to look at the distribution of the derived allele among populations in the world. The derived alleles of the core SNP and its linked SNPs, such as rs3751248, are mainly observed in Asian populations (Figure S4), supporting the possibility of local adaptations (e.g., adaptations specific to Asian populations). Including the target site, the candidate SNPs in the fifth region were located in *SYCP3* and its upstream region. *SYCP3* is involved in the pairing and crossover of homologous chromosomes during meiosis [62]. Such a function should directly affect fitness, so a beneficial mutation in this gene could be selected for. Fundamentally, its effect on fitness should not only be for individuals in Asia but for individuals everywhere. Therefore, we consider that the trait under selection may not be related to meiosis, and that this gene region may be related to functions that are not yet elucidated.

The second case is the core region of rs3789228 on *DCDC2*. This SNP was distinguished as the younger SNP to the core SNP of rs1091047, based on the global derived allele count. To date, there is no study investigating whether the derived allele of this younger SNP itself is risk or non-risk for dyslexia of Chinese characters, but the derived allele of the core SNP is a non-risk type [12]. Based on our analyses, the target site may be located in the first to second region, where both candidate SNPs showed *p* < 0.001. The second SNP (rs12055879) in this region is located in both the enhancer region and CTCF binding site; since this SNP may affect expression regulation, we speculate that it is the target site in this core region. Like in the case of rs17031962 on *GNPTAB*, even if the target site has some biological function, it is unknown which gene is affected by mutations at this site and which trait is affected. Therefore, if natural selection has acted, any of the three scenarios mentioned above would also be possible for this case (i.e., pleiotropy, genetic hitchhiking, and selection for prior functions explained by neuronal recycling hypothesis and cultural neural reuse).

Looking at the distribution of the derived alleles among populations in the world (Figure S4) and the descriptions of haplotypes in the core region using samples from all populations (Figure S5), we found that derived alleles of the core SNP (rs1091047) and its linked SNPs in frequency class 10 were carried by various haplotypes containing sequences from both African and non-African populations. Meanwhile, derived alleles of the younger SNP (rs3789228) and its linked SNPs, including the target site (rs12055879), in frequency class 9 were carried by a small number of haplotypes predominantly from non-African populations. Therefore, these derived alleles may have spread after out of Africa migration. The derived allele frequency of the target site (rs12055879) seemed to be higher in East Asian populations than in other non-African populations (Figure S4). Interestingly, according to previous studies, the derived allele of the core SNP (rs1091047) was the non-risk type in the Chinese population, whereas the derived allele was the risk type in the European ancestry population where people use an alphabetic language [12,70]. However, we cannot infer whether the mutation on the target site itself has an effect on a certain prior function related to the reading ability of Chinese characters or not, because the effect of mutations on this target site has also not been explored.

Although the present study did not investigate the relationship between allele distribution and writing systems, there are cases showing a correlation between human genetic variation and certain features of the spoken language. The frequency of an allele group of the READ1 regulatory element in *DCDC2* was found to be positively correlated with the number of consonants [71]. Moreover, the frequency of particular haplotypes of *ASPM* and *Microcephalin* in populations was found to be correlated with use of linguistic tone [72]. *ASPM* and *Microcephalin* are genes related to brain size, and it is arguable whether they have or have not been under positive selection for brain growth [73,74,75,76].

Distinct from these previous studies, we focused on examining whether natural selection has acted on the alleles of SNPs reported to be risk/non-risk for reading ability. While 2D SFS-based statistics suggested that two core regions could be under selective sweep, this was not supported by the results of *nSL*. Several reasons could be considered for this discrepancy. One of the possibilities is recombination rate variation, which should affect the haplotype length [45]. In the core region of rs17031962 on *GNPTAB*, $r^{2}$ values with the core SNP sharply declined, especially in the upstream side (i.e., the region with a smaller genomic position number). This implies that the core region could be located very close to a recombination hotspot, which would weaken the signal of selective sweep detected using *nSL*. Another possibility is that LD is broken down by recombination events over time, which renders it difficult to detect selection signals [45]. An SNP with a high derived allele frequency is assumed to have such a short LD. In addition, when the derived allele frequency is higher, the power of *nSL* declines in a subpopulation of structured populations [77], such as populations in 1 KG. Although we only showed the results of the 15 core SNPs for *nSL*, we found that the result of *nSL* for rs3789228 (the younger SNP for the core SNP of rs1091047 on *DCDC2*) was also not significant (normalized *nSL* = 0.1851; *p* = 0.427). The derived allele frequency of rs3789228 in EAS is 77.6%, and therefore, this frequency could be relatively too high for *nSL* to detect sweep signals.

In particular, we searched for the target site in two core regions, which could be under selective sweep. Our study supported the possibility of genetic hitchhiking: The target sites could have functional effects on genes other than dyslexia-related genes, *GNPTAB* and *DCDC2*. These effects are not biologically confirmed but were speculated based on annotation data. Future experiments are necessary to verify whether these target sites actually have a functional effect and which gene is affected. The findings in our study should be the results seen only in our study populations, i.e., EAS in 1 KG. In order to check sampling effects, follow-up studies are required when other East Asian data become available. In addition, although beyond our study, the validity of the association between core SNPs and reading ability needs to be confirmed by replications.

Modern society has introduced public education and demands universal literacy [9,28]. So, primarily, the environment of the modern society likely determines which allele is “risk” or “non-risk” for reading ability. Dyslexia should basically be a consequence of neutral variation. Even in the case where selection may have acted, the selected trait should be different from reading ability itself.

## Figures and Tables

**Table 1:** The single nucleotide polymorphisms (SNPs) associated with dyslexia of Chinese characters in previous studies.

Gene	Core SNP	Chr.	Position	Risk Allele	Derived Allele Frequency		References
			(GRCh37/hg19)		EAS	(EAS and KPGP)	
*KIAA0319L*	rs28366021	1	36,022,859	Ancestral	0.234	(0.227)	[15]
*ROBO1*	rs4535189	3	79,489,971	Derived	0.366	(0.373)	[14]
*DCDC2*	rs807724	6	24,278,869	Ancestral	0.957	(0.956)	[20]
*DCDC2*	rs1091047	6	24,295,256	Ancestral	0.817	(0.823)	[12]
*KIAA0319*	rs2760157	6	24,578,272	Ancestral	0.456	(0.470)	[21]
*KIAA0319*	rs807507	6	24,579,867	Derived	0.188	(0.187)	[21]
*KIAA0319*	rs4504469	6	24,588,884	Derived	0.112	(0.122)	[15]
*DOCK4*	rs2074130	7	111,487,098	Derived	0.101	(0.115)	[15]
*DRD2*	rs1079727	11	113,289,182	Derived	0.416	(0.420)	[22]
*GNPTAB*	rs17031962	12	102,146,558	Ancestral	0.294	(0.297)	[23]
*DYX1C1*	rs11629841	15	55,777,638	Derived	0.058	(0.056)	[24]
*DYX1C1*	rs3743205	15	55,790,530	Derived	0.035	(0.037)	[25]
intergenic region	rs8049367	16	3,980,445	Derived	0.339	(0.340)	[26]
*NAGPA*	rs882294	16	5,092,118	Derived	0.189	(0.188)	[23]
*DIP2A*	rs2255526	21	47,971,539	Derived	0.264	(0.262)	[27]

**Table 2:** The results of *nSL* for the core SNPs.

Gene	Core SNP	Normalized *nSL*	*p*-Value
*KIAA0319L*	rs28366021 ^a^	0.0771	0.469
*ROBO1*	rs4535189	−0.1882	0.575
*DCDC2*	rs807724	1.1328	0.129
*DCDC2*	rs1091047	−0.5967	0.725
*KIAA0319*	rs2760157	−2.1853	0.986
*KIAA0319*	rs807507	0.7329	0.232
*KIAA0319*	rs4504469	0.7098	0.239
*DOCK4*	rs2074130	0.3068	0.379
*DRD2*	rs1079727	−0.1744	0.569
*GNPTAB*	rs17031962	1.2369	0.108
*DYX1C1*	rs11629841	−0.0922	0.537
*DYX1C1*	rs3743205	−0.1939	0.577
intergenic region	rs8049367	−0.4421	0.671
*NAGPA*	rs882294	0.2399	0.405
*DIP2A*	rs2255526	-	-

^a^ Representative for rs28366021.

**Table 3:** $F_{c}$ statistic results for the core SNPs and three younger SNPs.

Gene	Core SNP	Number of Derived Alleles n = 1008	Length of the Core Region	Number of Segregating Sites	$F_{c}$	*p*-Value
*KIAA0319L*	rs28366021	236	330,223	2204	0.1476	0.718
*ROBO1*	rs4535189	369	124,626	866	0.1287	0.316
*ROBO1*	rs73129039 ^a^	363	124,626	866	0.1232	0.303
*DCDC2*	rs807724	965	5910	53	0.6742	0.159
*DCDC2*	rs1091047	824	41,134	334	0.3044	0.111
*DCDC2*	rs3789228 ^b^	782	41,134	334	0.2020	0.068 *
*KIAA0319*	rs2760157	460	7387	53	0.7765	0.939
*KIAA0319*	rs807507	189	11,475	81	0.0220	0.111
*KIAA03219*	rs4504469	113	32,025	241	0.0736	0.529
*DOCK4*	rs2074130	102	-	-	-	-
*DRD2*	rs1079727	419	38,525	372	0.1370	0.260
*GNPTAB*	rs17031962	296	136,804	868	0.0400	0.038 *
*DYX1C1*	rs11629841	58	130,280	1113	0.0589	0.769
*DYX1C1*	rs3743205	35	242,254	2024	0.0680	0.963
*DYX1C1*	rs79024225 ^c^	31	242,254	2024	0.0308	0.758
intergenic region	rs8049367	342	14,513	177	0.1486	0.428
*NAGPA*	rs882294	191	34,706	339	0.2875	0.905
*DIP2A*	rs2255526	266	67,101	661	0.0899	0.361

* *p* < 0.1; ^a^ the younger SNP of rs4535189 on *ROBO1*; ^b^ the younger SNP of rs1091047 on *DCDC2*; ^c^ the younger SNP of rs3743205 on *DYX1C1*.

## Supplementary Materials

The following are available online at https://www.mdpi.com/2073-4425/11/6/658/s1, Figure S1: $r^{2}$ with the core SNP in 1-Mb region, Figure S2: Haplotypes observed in EAS for the core region of rs17031962, Figure S3: Extraction of rs17031962 and its linked SNPs, Figure S4: Derived allele frequencies of the 15 core SNPs, Figure S5: Haplotypes observed in the core region of rs3789228.
