# Supplementary material for: Do Genes Associated with Dyslexia of Chinese Characters Evolve Neutrally?
Source: Genes (Basel). 2020 Jun 17;11(6):658. doi: 10.3390/genes11060658 (PMC7349701; doi:10.3390/genes11060658)
Supplement: Supplementary file 1 [file genes-11-00658-s001.pdf]

Supplementary Figures

Do genes associated with dyslexia of Chinese characters evolve neutrally?

Kumiko V. Nishiyama, Yoko Satta and Jun Gojobori

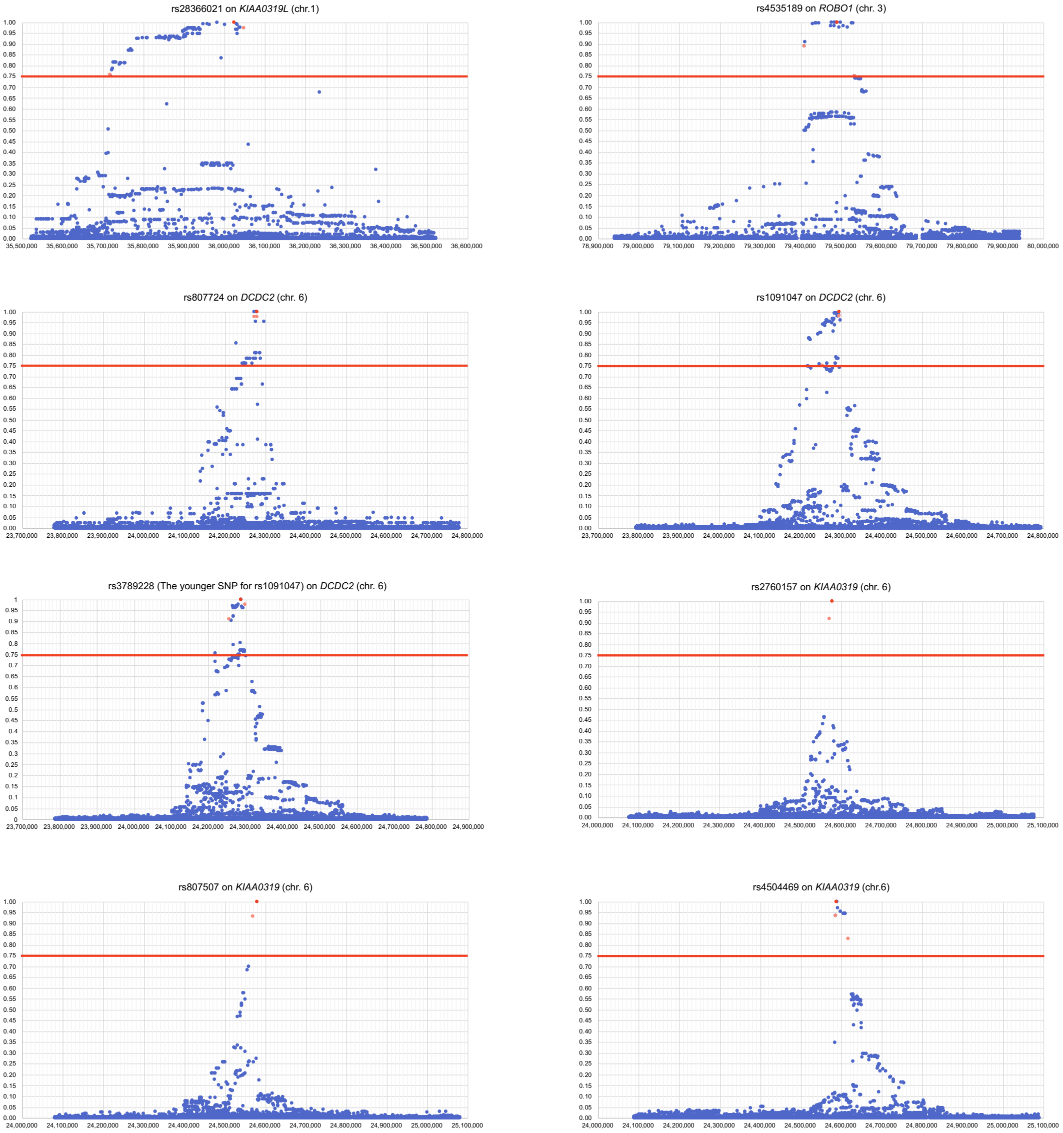

Figure S1 r^2 with the core SNP in a 1-Mb region, where the core SNP is centered (red dot). Pink dots represent SNPs that are boundaries of core regions. In some cases, core SNPs themselves are one of the boundaries. Red line represents $r^2 = 0.75$. The SNP is not taken as the boundary in the case when a derived allele at the core SNP is linked to ancestral alleles in the linked SNPs, and when ancestral allele at the core SNP is linked to derived alleles in linked SNPs, even if $r^2 > 0.75$. The surrounding region of rs2255526 on *DIP2A* is less than 1-Mb because the core SNP is located near the end of chromosome 21.

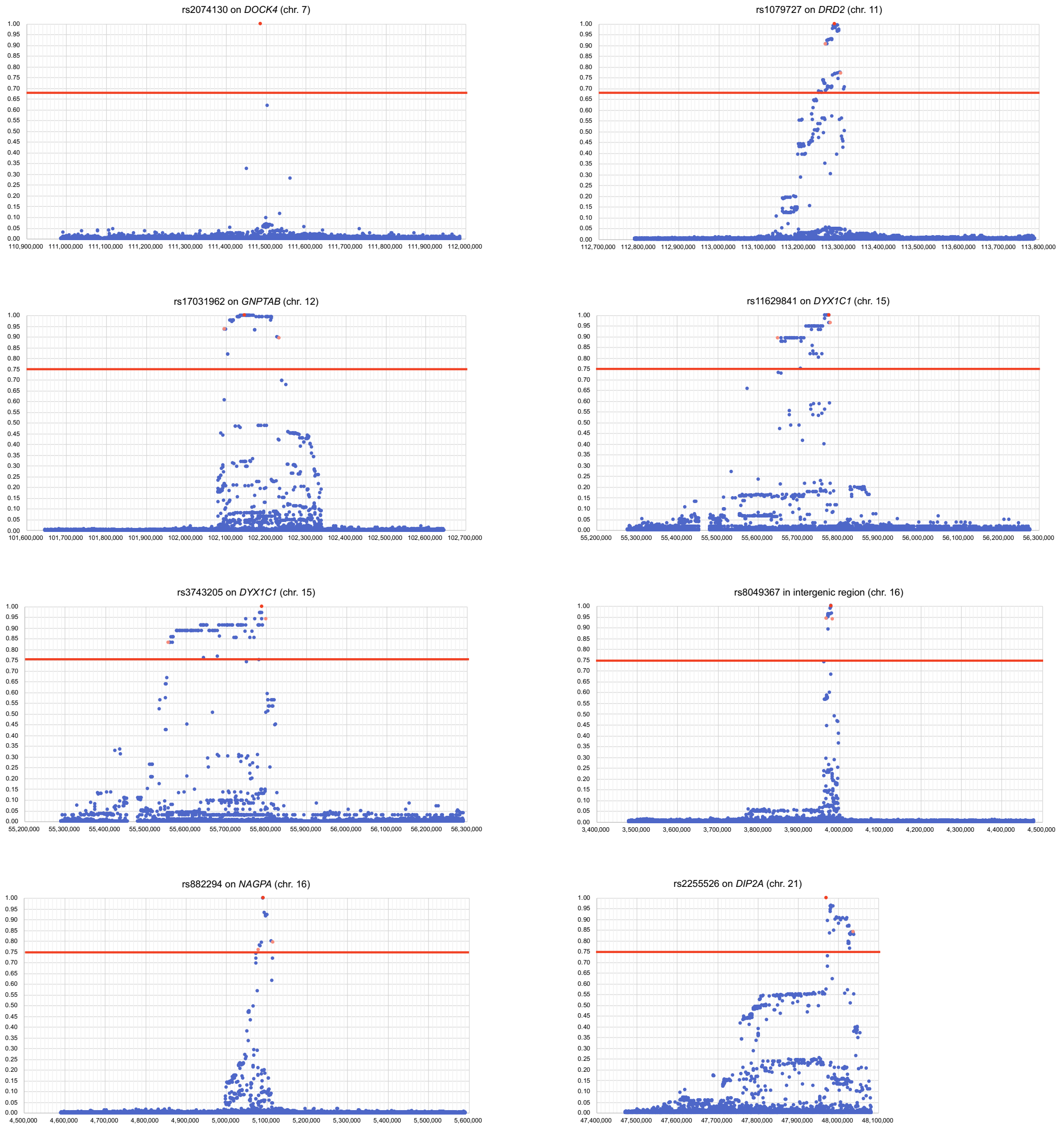

Figure S1 (Continued)

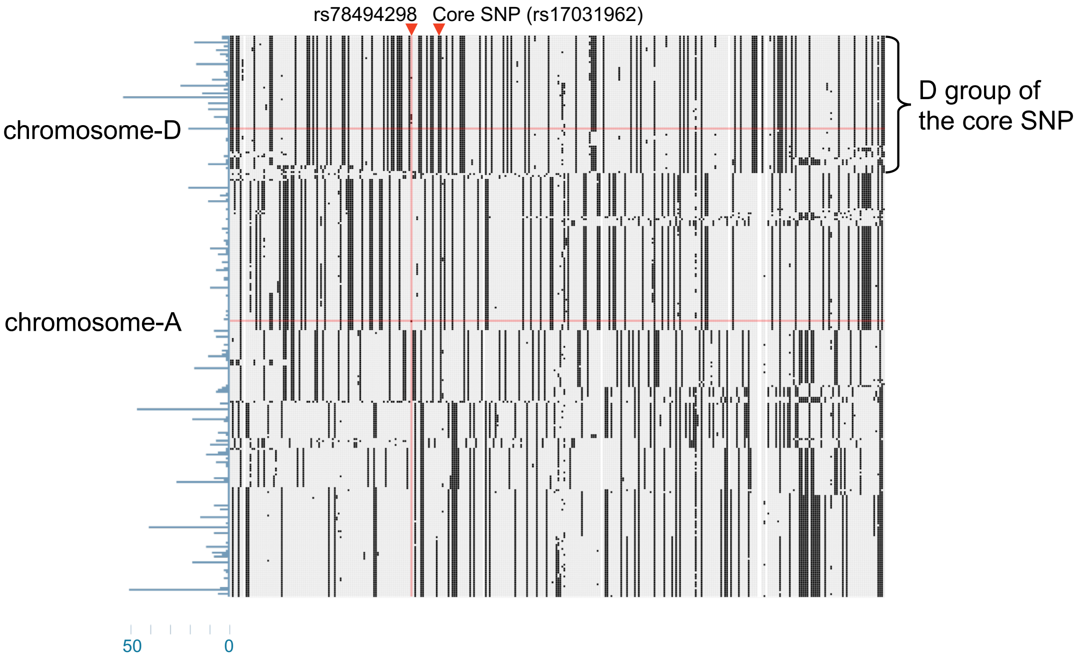

Figure S2 Haplotypes observed in EAS for the core region of rs17031962, using SNPs with minor allele frequency (MAF) in EAS ≥ 0.01. Columns represent genomic positions, and rows represent haplotypes. Haplotypes were sorted according to neighbor joining tree constructed by MEGA7 [1]. For each haplotype, a black or grey cell represents derived or ancestral allele, respectively, at the position. The positions of rs17031962 and rs78494298 are indicated by red triangles. The vertical red line indicates the position of rs78494298. The lengths of blue bars on the left side display the counts of each haplotype. "chromosome-D" indicates the one chromosome of HG00707 carrying the derived allele of the core SNP, and "chromosome-A" indicates the other chromosome of HG00707 carrying the ancestral allele of the core SNP. Horizontal red lines represent haplotypes containing each of the two chromosomes of HG00707.

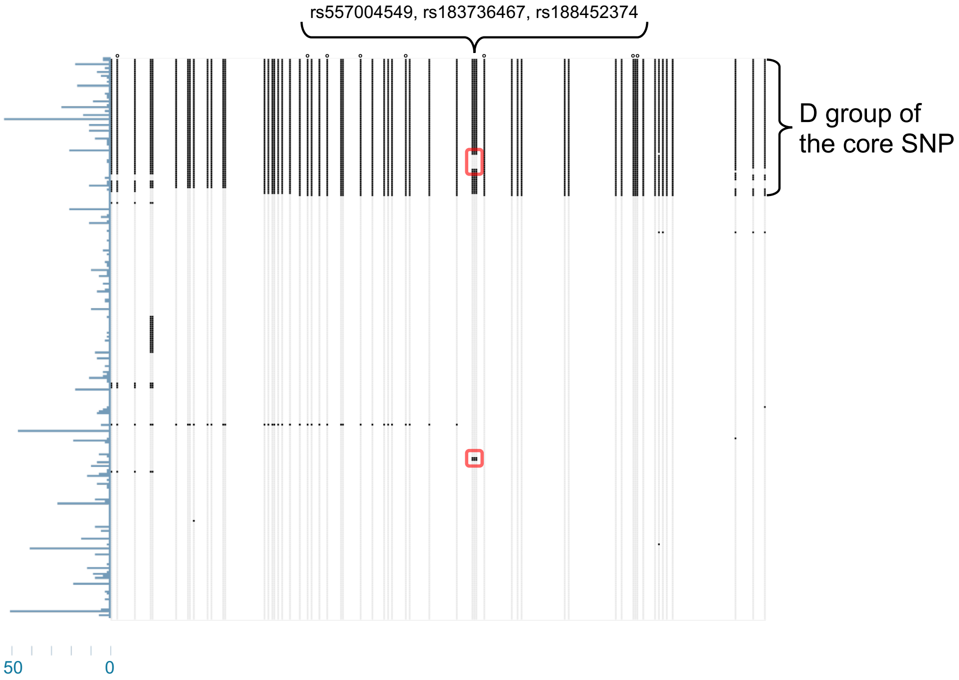

Figure S3 rs17031962 and its linked SNPs. SNPs with "o" above indicate linked SNPs in globally older frequency classes than rs17031962, which were ignored in the analysis. Three consecutive SNPs (rs557004549, rs183736467, rs188452374), which were excluded from the analysis, are noted. Different pattern from the other linked SNPs are marked with red rectangles: some of their derived alleles seemed to be linked to ancestral alleles of the other candidate SNPs, and vice versa.

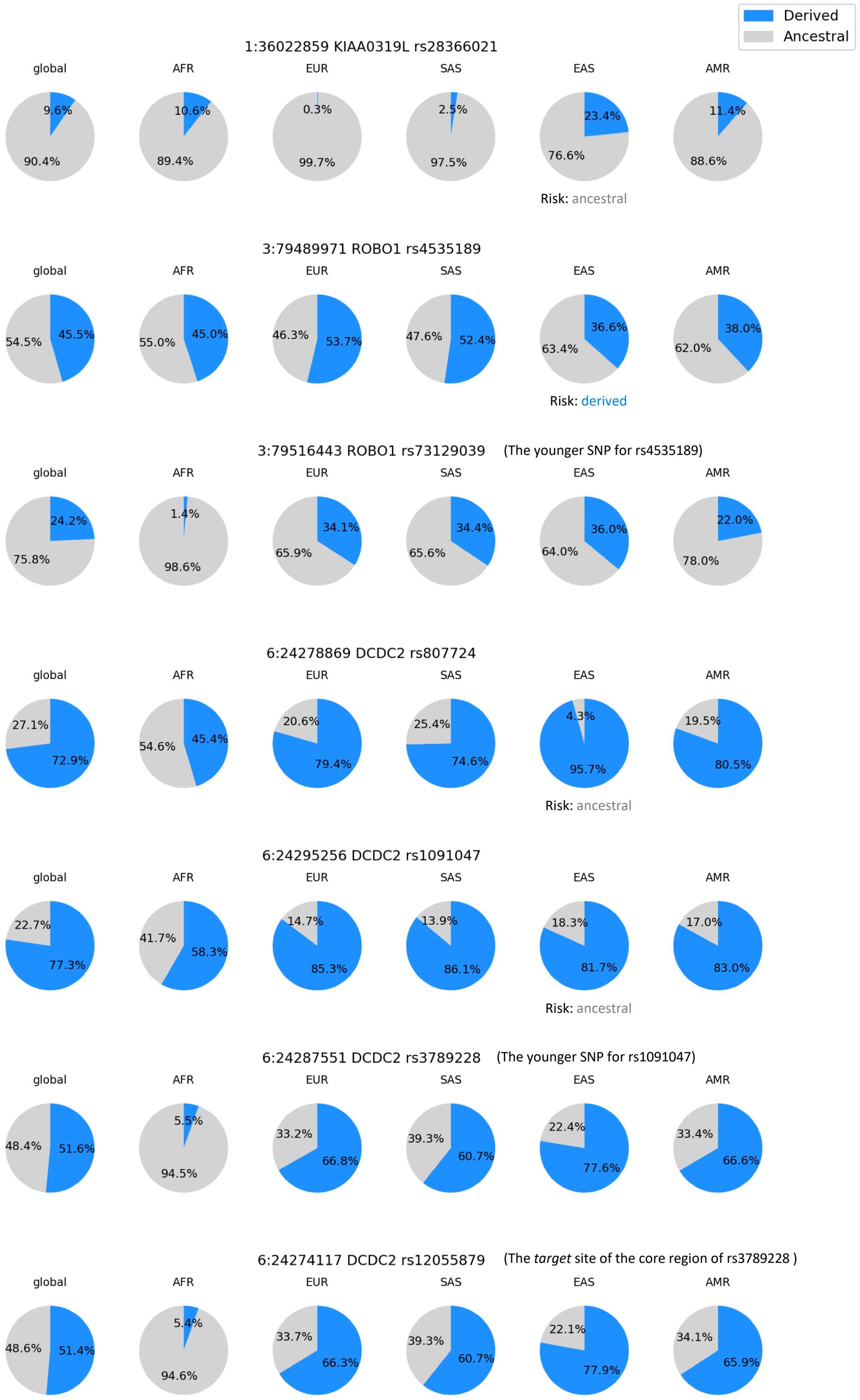

Figure S4 The derived allele frequencies of the 15 core SNPs in global, African (AFR), European (EUR), South Asian (SAS), East Asian (EAS), and American (AMR) populations of 1KG. Three younger SNPs and two target sites are also displayed after their core SNPs. For the 15 core SNPs, the risk alleles reported in the previous studies in Table 1 are noted below the pie charts of EAS.

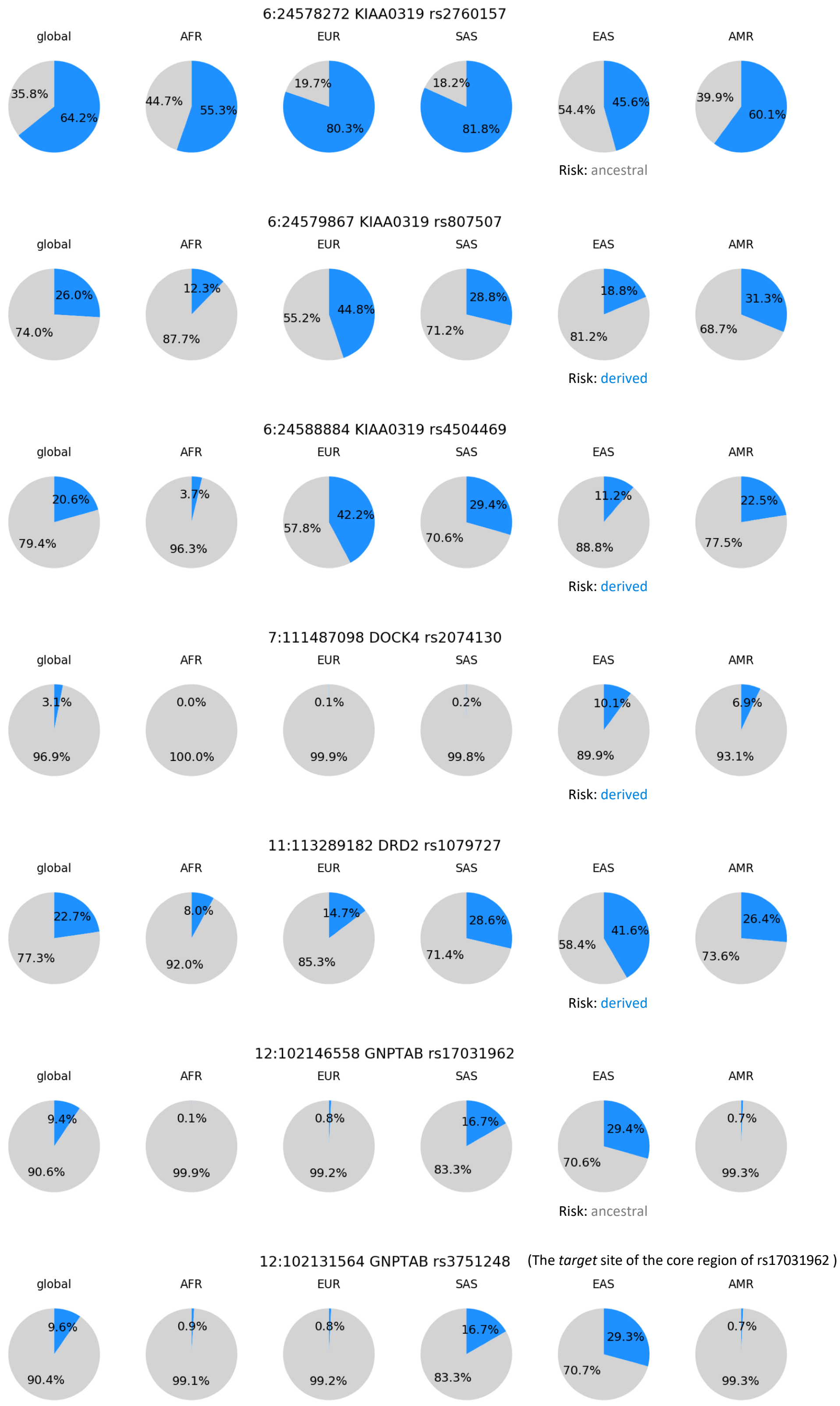

Figure S4 (Continued)

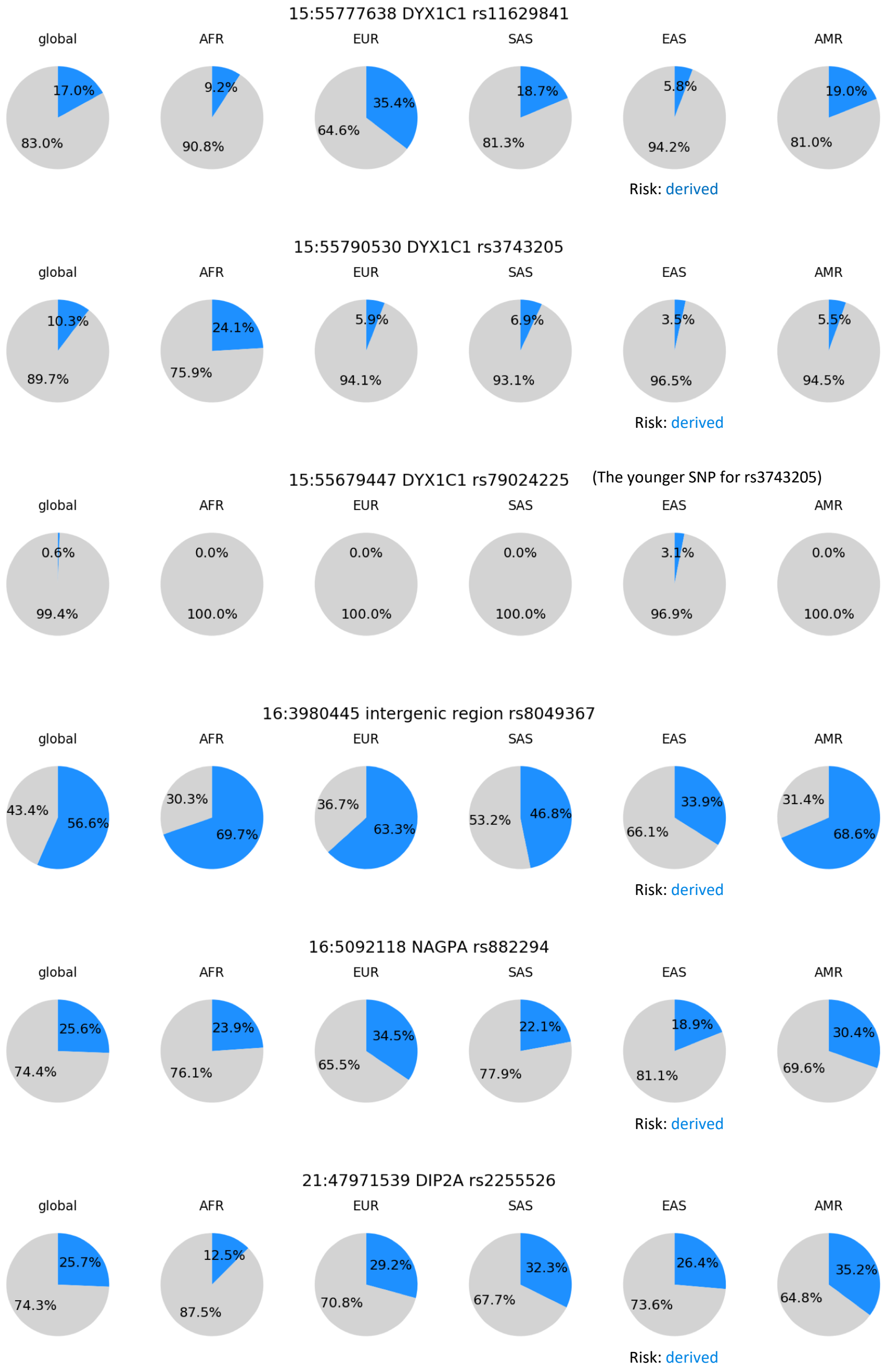

Figure S4 (Continued)

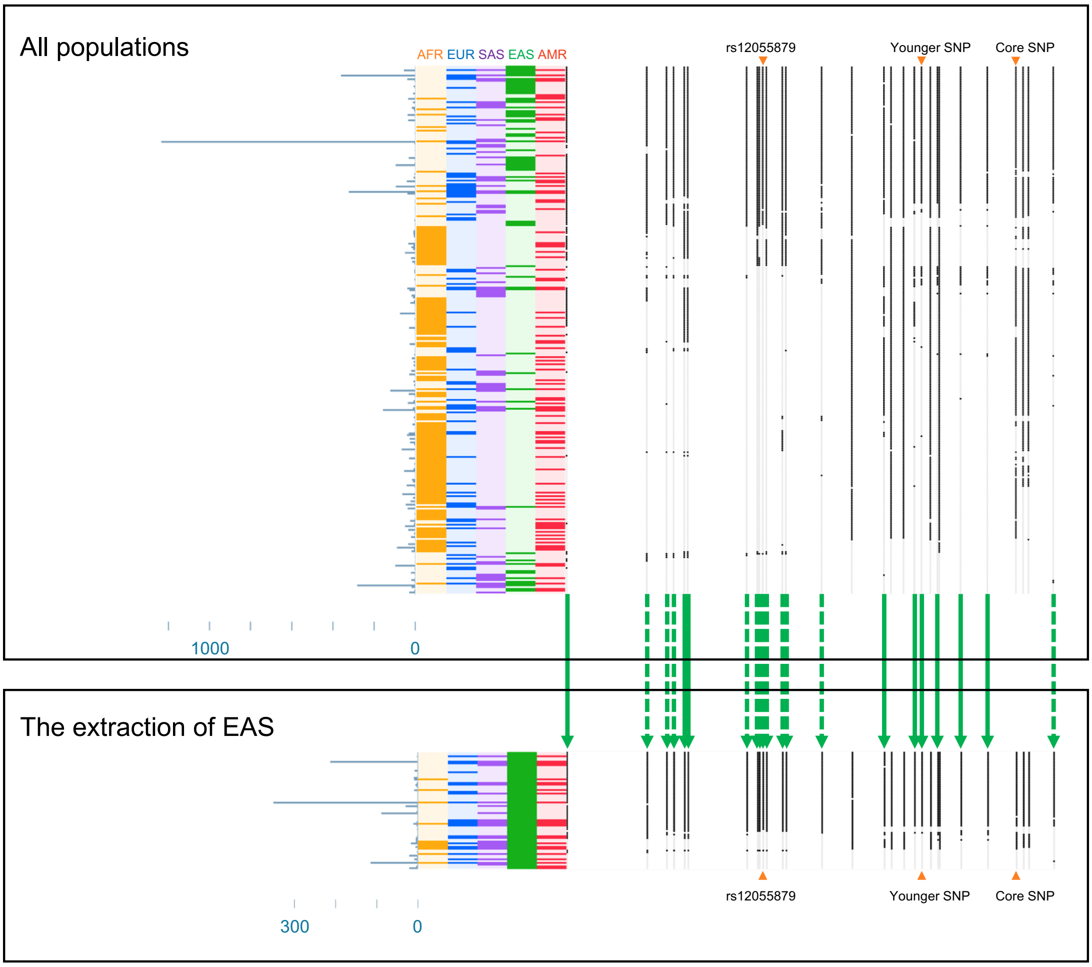

Figure S5 Top: haplotypes observed in all populations of 1KG for the core region of rs3789228, defined by SNPs with global MAF ≥ 0.005. Columns represent genomic positions, and rows represent haplotypes. Haplotypes were sorted according to neighbor joining tree constructed by MEGA7 [1]. For each haplotype, a black or grey cell represents derived or ancestral allele, respectively, at the position. Only rs3789228 and rs1091047 and their linked SNPs are indicated. Bottom: haplotypes observed in EAS. SNPs with solid green arrows indicate SNPs linked to both rs3789228 and rs1091047 in global frequency class 9; SNPs with broken green arrows indicate SNPs linked only to rs3789228 in global frequency class 9; SNPs with no arrows indicate linked SNPs in global frequency class 10. The five colored columns represent presence/absence of sequences from the five populations of 1KG for each haplotype. In the colored columns, dark tone indicates at least one sequence from the population is present in the haplotype while light color indicates no sequence from the population is observed in the haplotype. The blue bar lengths on the left side indicate the counts of each haplotype.

References

1. Kumar, S.; Stecher, G.; Tamura, K. MEGA7: Molecular Evolutionary Genetics Analysis Version 7.0 for Bigger Datasets. *Mol. Biol. Evol.* **2016**, *33*, 1870–1874.
